# Suppression of VEGF and inflammatory cytokines, modulation of Annexin A1 and organ functions by galloylquinic acids in breast cancer model

**DOI:** 10.1038/s41598-023-37654-4

**Published:** 2023-07-28

**Authors:** Mohamed Abd El-Salam, Ghada El-Tanbouly, Jairo Bastos, Heba Metwaly

**Affiliations:** 1 Department of Pharmacognosy, Faculty of Pharmacy, Delta University for Science and Technology, Gamasa, 11152 Egypt; 2 School of Pharmacy and Biomolecular Sciences, Royal College of Surgeons in Ireland, Dublin, D02 VN51 Ireland; 3 Department of Pharmacology, Faculty of Pharmacy, Delta University for Science and Technology, Gamasa, 11152 Egypt; 4 Department of Pharmaceutical Sciences, School of Pharmaceutical Sciences of Ribeirão Preto, University of São Paulo, Ribeirão Preto, 14040-900 Brazil; 5 Department of Pharmaceutical Biochemistry, Faculty of Pharmacy, Alexandria University, Alexandria, 21500 Egypt

**Keywords:** Cancer, Drug discovery

## Abstract

The ongoing development of novel drugs for breast cancer aims to improve therapeutic outcomes, reduce toxicities, and mitigate resistance to chemotherapeutic agents. Doxorubicin (Dox) is known for its significant side effects caused by non-specific cytotoxicity. In this study, we investigated the antitumor activity of galloylquinic acids (BF) and the beneficial role of their combination with Dox in an Ehrlich ascites carcinoma (EAC)-bearing mouse model, as well as their cytotoxic effect on MCF-7 cells. The EAC-mice were randomized into five experimental groups: normal saline, Dox (2 mg/kg, i.p), BF (150 mg/kg, orally), Dox and BF combined mixture, and a control group. Mice were subjected to a 14-day treatment regimen. Results showed that BF compounds exerted chemopreventive effects in EAC mice group by increasing mean survival time, decreasing tumor volume, inhibiting ascites tumor cell count, modulating body weight changes, and preventing multi-organ histopathological alterations. BF suppressed the increased levels of inflammatory mediators (IL-6 and TNF-α) and the angiogenic marker VEGF in the ascitic fluid. In addition, BF and their combination with Dox exhibited significant cytotoxic activity on MCF-7 cells by inhibiting cell viability and modulating Annexin A1 level. Moreover, BF treatments could revert oxidative stress, restore liver and kidney functions, and normalize blood cell counts.

## Introduction

Cancer remains the main cause of death worldwide. To date, medical research continues to face challenges in managing cancer burden^1^. Breast cancer is the most prevalent cause of cancer-associated mortality among women globally. According to the American Cancer Society statistics 2022, breast, lung and colorectal cancers account for 51% of all new diagnoses in women, with breast cancer alone accounting for almost one-third of diagnosed cases^2^. The disturbance in cell growth and death homeostasis, oxidant/antioxidant balance, massive release of reactive oxygen species (ROS)-induced oxidative stress, and the subsequent cellular damage of lipids, proteins, and DNA, all have been implicated in the pathogenesis of cancer^3^.

Ehrlich ascites carcinoma (EAC) is a murine spontaneous breast cancer model widely employed to explore different aspects of tumorigenesis and evaluate the efficacy of anticancer agents. It is well-known for its high sensitivity to medications and its resemblance to human breast tumors. EAC can rapidly grow in the peritoneum of animal models, leading to a significant increase in ascitic volume and cell numbers, mimicking the characteristics of the original human breast tumor^4^.

**Figure 1:**
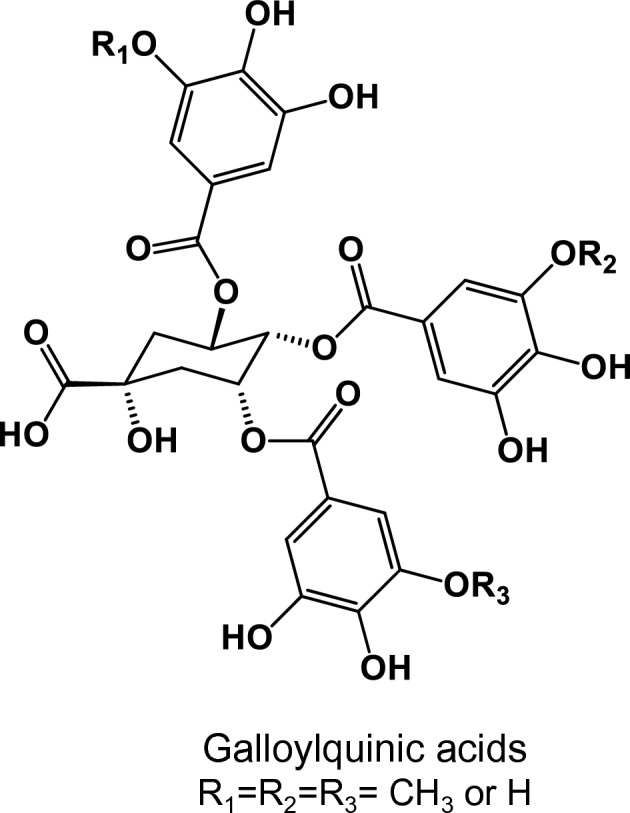
Chemical structure of galloylquinic acid compounds extracted from the* n*-butanolic fraction (BF) of *Copaifera lucens* leaves.

Doxorubicin (Dox) is a widely utilized first-line therapy for breast cancer. It exerts its anticancer effects through various mechanisms, such as disrupting DNA structure and impeding the generation of free radicals. However, the efficacy of Dox is often hindered by multi-drug resistance, as well as its potential to induce toxicities in multiple organs including the liver, kidneys, lungs, and heart. These limitations pose challenges to the effective use of Dox in breast cancer treatment^5,6^. Therefore, many studies have been focused on exploring bioactive natural agents that can prohibit, delay, or reverse multistage of breast cancer progression. Extensive research has been conducted on various phytochemicals, including Quercetin, concerning their potential impact on breast cancer. One notable outcome of these studies is the significant reduction observed in tumor volume in Ehrlich solid tumor-bearing mice treated with Quercetin^7^. Medicinal plants found in tropical regions, such as Brazil, are abundant sources of crucial compounds for treating various diseases, particularly in developing countries. Different species of Copaifera, including *C. lucens*, *C. langsdorffii*, and *C. multijuga*, are widely distributed in Brazil. Galloylquinic acids (Fig. 1) are polyphenolic compounds known as the major secondary metabolites found in the *n*-butanolic (BF) and aqueous fractions of Copaifera leaf extracts. These compounds hold significant therapeutic potential^8^. Our previous studies have reported that galloylquinic acid compounds extracted from Copaifera leaves are mainly responsible for their antiurolithic activity^9^ and gastroprotective effect against gastric ulcer, anti-candidiasis^10^, as well as cytoprotective effect against gastric adenocarcinoma cells^11^. Moreover, a previous study has reported their preventive effect against colorectal cancer and DNA damage in rats^12^.

Recent research studies have revealed the potential antioxidant activity of these polyphenolic compounds, indicating their effectiveness in lowering the risk of breast cancer development^9,11^.

Toxicological evaluations of Copaifera extracts have substantiated the absence of genotoxicity and adverse effects on normal renal function, thus establishing a favorable low toxicity profile with an LD_50_ value surpassing 2000 mg/kg^13,14^. Hence, the robust safety profile exhibited by Copaifera extracts has instigated our research inquiry into their prospective role in breast cancer chemoprevention and their combinatorial therapeutic application alongside Dox. Angiogenesis plays a crucial role in the progression and advancement of tumors in different cancer types, including breast cancer, and is closely linked to poor patient survival rates. Vascular endothelial growth factor (VEGF) serves as a pivotal mediator in the process of angiogenesis^15^. Several recent studies have highlighted the significance of vascular endothelial growth factor (VEGF) in breast cancer, linking it to pathogenesis, metastasis, and potential resistance to anti-hormonal and chemotherapeutic treatments^16,17^. Consequently, targeting angiogenic factors, particularly VEGF inhibitors, presents a promising approach to combat breast cancer progression and enhance patient survival rates^16^. Nevertheless, the issue of drug resistance remains a major challenge in the field of angiogenesis-targeted therapy^18^. El Bakary et al.^20^ revealed the identification of the natural substances; bee venom and its primary component, melittin, as antiangiogenic agents following radiation exposure in EC-bearing mouse model. As a result, bee venom and melittin could potentially serve as a therapeutic approach to enhance the radiation response of solid tumors^19^. The authors also employed the same animal model to demonstrate the immune-stimulating outcome of Chrysin, a natural flavone present in honey and propolis, in combination with γ-irradiation^20^.

Previous studies documented the association between release of inflammatory mediators and cancer. Tumor necrosis factor-alpha (TNF-α) is involved in tumor growth, angiogenesis and metastasis in EAC model^21^. In addition, interleukin (IL)-6 and prostaglandin E2 (PGE2) expressions were elevated in EAC and human ascetic tumors^22,23^. Inflammatory PGE2 expression is the result of overactivation of arachidonic acid pathway through the action of phospholipase A2 enzyme. Elevated levels of prostaglandin E2 (PGE2) have been implicated in various detrimental effects, including immunosuppression, promotion of neoangiogenesis, stimulation of tumor cell growth, progression, metastasis, and overall survival^24^. Interestingly, inhibitors targeting this pathway have been shown to exert potent antitumor effects in animal models and clinical trials^25,26^.

Annexin A1 (ANXA1), also known as lipocortin-1, is an endogenous glucocorticoid-regulated protein that plays a crucial role in counterbalancing inflammation and restoring homeostasis. It suppresses phospholipase A2, regulates several inflammatory mediators including cyclooxygenase-2 (COX-2) and inducible nitric oxide synthase (iNOS), reduces proinflammatory cytokines, promotes neutrophil apoptosis, and inhibits neutrophil infiltration and accumulation^27,28^. However, cancer cells often employ compensatory mechanisms to promote their survival, thereby reducing the efficacy of anti-neoplastic treatments. To overcome this challenge, a combination therapy approach targeting multiple pathways simultaneously could be employed to enhance the therapeutic effect. By modulating various pathways, the use of multiple drugs can potentially exert a more potent anticancer action.

This work aimed to investigate the potential of BF compounds as chemo-preventive agents with minimal adverse effects in two breast cancer models: the murine EAC model in vivo and the MCF-7 cell model in vitro*.* Additionally, the efficacy of a multimodal therapy combining BF extract and the chemotherapeutic drug Dox was examined. Furthermore, our study explored the anti-angiogenic and anti-inflammatory effects of these compounds on breast cancer progression, along with their impact on cancer cell growth, immune cell infiltration, tumor cytokine levels, oxidative stress, ANXA1 level and the restoration of multi-organ functions.

## Results

### Inhibition of tumor growth by BF, Dox, and their combination: effects on survival time, body weight, and tumor size in EAC-bearing mice

**Figure 2:**
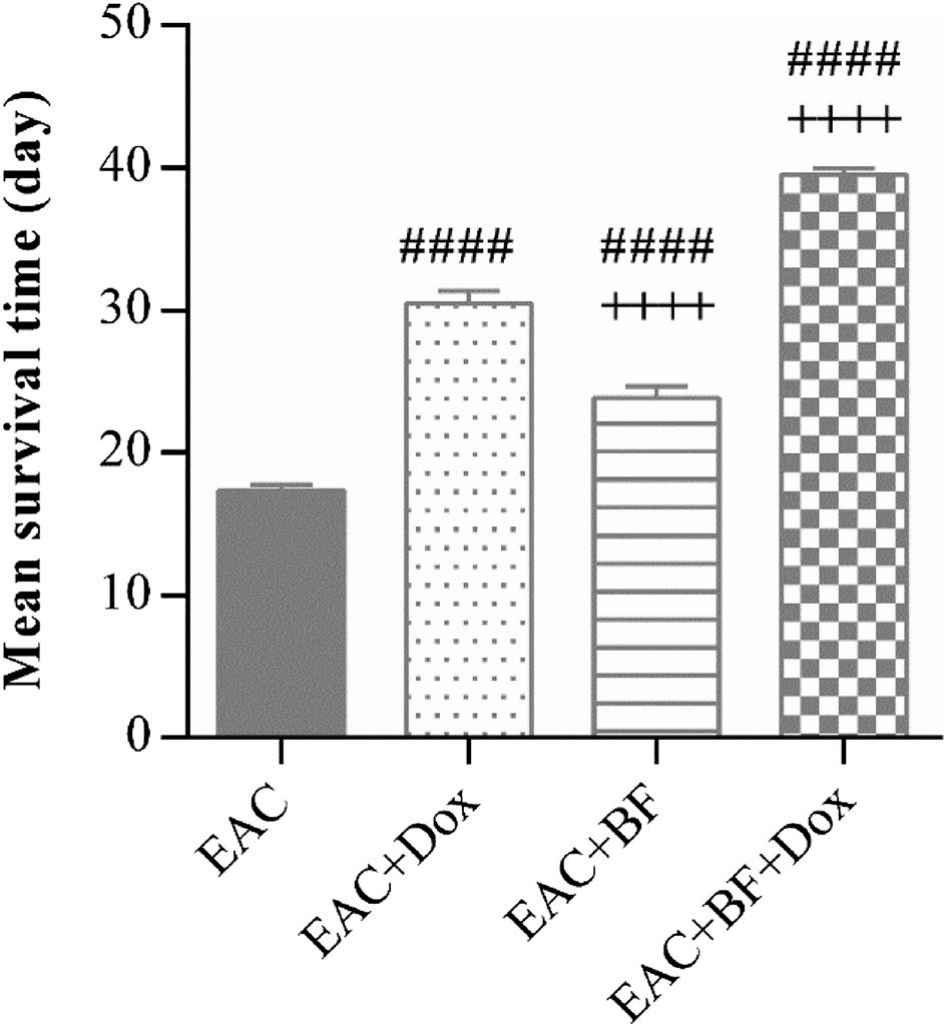
Effect of BF, Dox, and their combination on mean survival time (MST) in Ehrlich ascites carcinoma (EAC)-bearing mouse model. Animals were treated with galloylquinic acids from the *n*-butanolic fraction of *C. lucens* (BF) (150 mg/kg, orally), Doxorubicin (Dox) (2 mg/kg, i.p.), or their combination for a duration of 14 days. Subsequently, the mean survival time of the mice was calculated. The data presented are expressed as the mean ± SE. Statistical significance at *p* < 0.001 is denoted by “####” in comparison to the EAC group, while “++++” indicates significance compared to the Dox-treated group.

**Figure 3:**
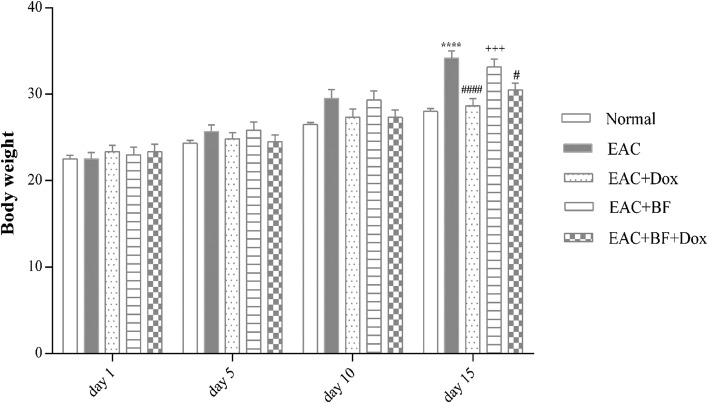
Impact of BF, Dox, and their combination on average body weight (in grams) of Ehrlich ascites carcinoma (EAC)-bearing mice. The effect of galloylquinic acids extracted from the *n*-butanolic fraction of *C. lucens* (BF) (150 mg/kg, orally), Doxorubicin (Dox) (2 mg/kg, i.p.), and their combination on the body weight of animals was monitored over a period of 15 days, with measurements taken every 5 days. The results were compared to a normal control group and EAC-bearing mice. The data presented represent the mean body weight ± SE. Significance values are indicated as follows: “****” indicates significance compared to the normal control group at *p* < 0.0001, “#” and “####” indicate significance compared to the EAC group at *p* < 0.05 and *p* < 0.0001, respectively, and “+++” indicates significance compared to the Dox-treated group at *p* < 0.001.

Antitumor effects were assayed in the ascetic fluid via recording the mean survival time (MST) and body weight changes illustrated in Figs. 2, and 3, respectively, in addition to immediate determination of tumor size markers: ascites tumor volume, viable and non-viable tumor cell count as shown in Table 1. The MST of mice increased in all treated groups with Dox, BF or their combination, compared to the untreated EAC group. Interestingly, the combination treated group recorded the highest survival, indicating that BF showed significant synergistic effect upon combining with Dox (Fig. 2). Furthermore, on day 15, Dox, and combination groups markedly reduced the EAC-induced elevation in body weight, while BF group showed no significant change in body weight compared to EAC group (Fig. 3).

**Table 1:** Effect of BF, Dox, and their combination on tumor size parameters (volume and cell count) in EAC-bearing mice.

	EAC	EAC + Dox	EAC + BF	EAC + BF + Dox
Viable cell count	83.34 ± 1.74	22.58 ± 2.45^**####**^	54.77 ± 2.21^**#### ++++**^	14.16 ± 1.67^**#### +**^
Non viable cell count	14.74 ± 1.60	77.75 ± 2.38^**####**^	45.32 ± 2.298^**#### ++++**^	84.17 ± 2.48^**####**^
Tumor volume	8.650 ± 0.37	2.633 ± 0.29^**####**^	5.133 ± 0.16^**#### ++++**^	1.350 ± 0.21^**#### +**^

Tumour inhibitory effects of galloylquinic acid compounds (BF) at a dose of 150 mg/kg (orally), doxorubicin (Dox) at a dose of 2 mg/kg (i.p.), and their combination were evaluated by measuring tumor cell count and volume in Ehrlich ascites carcinoma (EAC) bearing mice. The data are presented as the mean ± standard error (SE). Statistical significance was determined as follows: #### *p* < 0.0001 versus untreated EAC group; +, ++++*p* < 0.05, 0.0001 versus Dox-treated group.

Meanwhile, all treated groups significantly inhibited the cell viability and tumor volume of EAC, in addition, the combined treatment of BF with Dox was the most potent (Table 1).

### Effect of BF, Dox, and their combination on hematological and biochemical parameters

**Figure 4:**
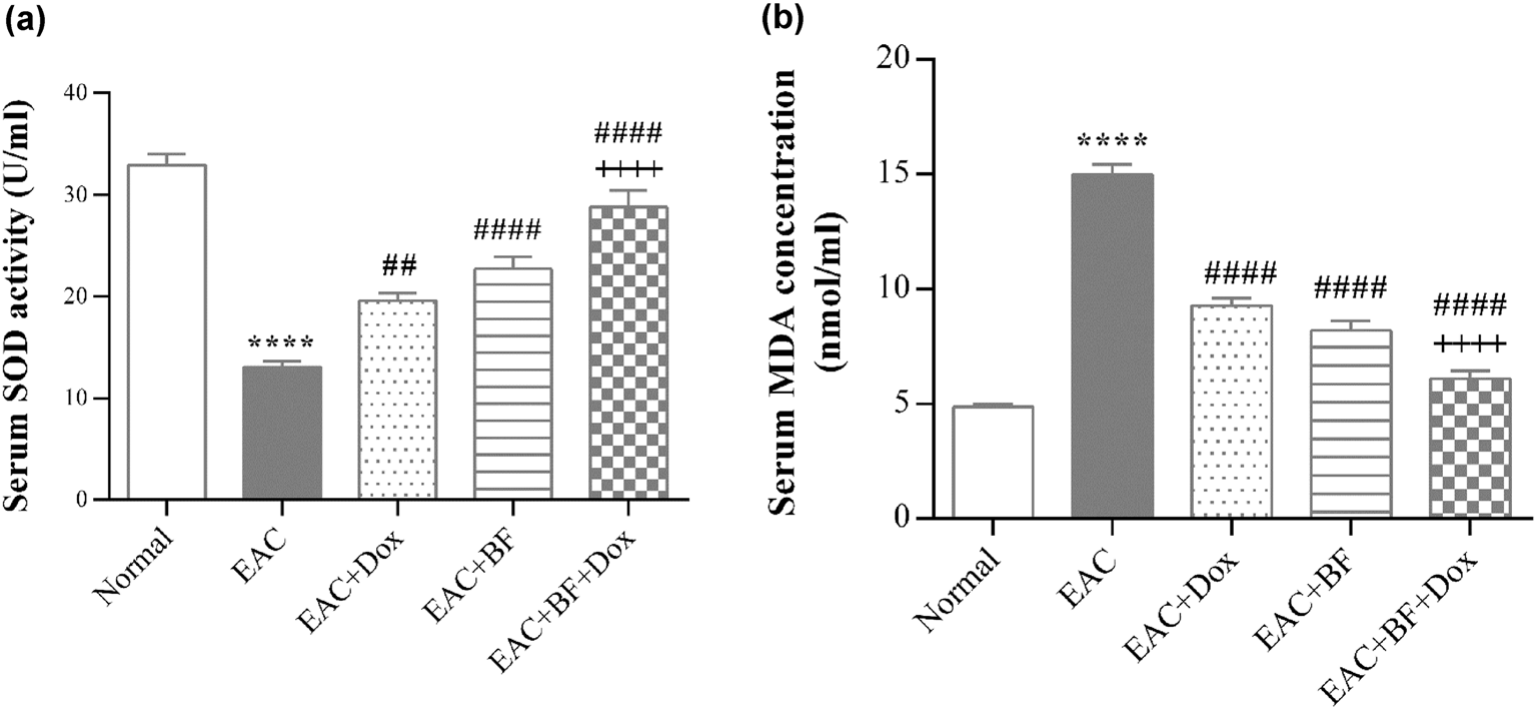
Effect of BF, Dox, and their combination on serum oxidative stress in Ehrlich ascites carcinoma (EAC)-bearing mice. Serum superoxide dismutase (SOD) activity (**a**) and levels of malondialdehyde (MDA) (**b**) were assessed colorimetrically in all studied groups: normal group, untreated EAC group, and groups treated with galloylquinic acids extracted from the* n*-butanolic fraction of *C. lucens* (BF) (150 mg/kg, orally), Doxorubicin (Dox) (2 mg/kg, i.p.), and their combined mixture after 14 days of treatment. The data presented represent the mean ± SE. Significance values are indicated as follows: “****” denotes *p* < 0.0001 compared to the normal control group, “##” and “####” denote* p* < 0.01 and *p* < 0.0001, respectively, compared to the untreated EAC group, and “++++” denotes* p* < 0.0001 compared to the Dox-treated group.

The hematological analysis showed a significant decrease in hemoglobin (Hb) levels in the untreated EAC group compared to the normal group. However, there was a remarkable restorative increase in Hb levels in all the treated groups with Dox, BF, and their combination. Furthermore, all treated groups showed a restoration of blood cell counts (RBCs and WBCs) towards normal levels. Among them, the combination treatment exhibited the most potent effect in restoring the blood cell counts compared to the group treated with Doxorubicin alone (Table 2). As shown in Fig. 4a, serum SOD activity was reduced and serum MDA level was elevated (Fig. 4b) in untreated EAC group, compared to normal control group. Meanwhile, all treated groups exerted a significant increase in SOD activity and a remarkable decrease in MDA level. Furthermore, the combined treatment of BF with Dox demonstrated the highest potency and resulted in a significant effect compared to the group treated with Dox alone (Fig. 4), indicating that the combination of BF compounds with Dox exhibited a significant synergistic effect on biochemical and hematological analyses.

**Table 2:** Hematological parameters in EAC-bearing mice treated with BF, Dox, and combination therapy.

	Normal	EAC	EAC + Dox	EAC + BF	EAC + BF + Dox
Hb	12.56 ± 0.2	7.890 ± 0.09****	10.55 ± 0.24^####^	11.20 ± 0.22^####^	11.68 ± 0.30^#### +^
RBC_S_	6.952 ± 0.16	4.158 ± 0.09****	5.582 ± 0.20^####^	6.090 ± 0.16^####^	6.583 ± 0.19^#### ++^
WBCs	6.737 ± 0.36	17.65 ± 0.46****	11.08 ± 0.55^####^	14.70 ± 0.24^## ++++^	8.265 ± 0.59^#### ++^

Effects of galloylquinic acid compounds (BF) at a dose of 150 mg/kg (orally), doxorubicin (Dox) at a dose of 2 mg/kg (i.p.), and their combination on hematological parameters were evaluated in Ehrlich ascites carcinoma (EAC)-bearing mice. Hemoglobin (Hb) levels, Red blood cell (RBC) counts, and White blood cell (WBC) counts were measured. Data are presented as mean ± standard error (SE). Statistical analysis revealed the following significance levels: **** *p* < 0.0001 versus normal control; ##, #### *p* < 0.01, 0.0001 versus untreated EAC group; and +, ++, ++++*p* < 0.05, 0.01, 0.0001 versus Dox-treated group.

### Effects of BF, Dox, and their combination on inflammatory markers and angiogenesis

**Figure 5:**
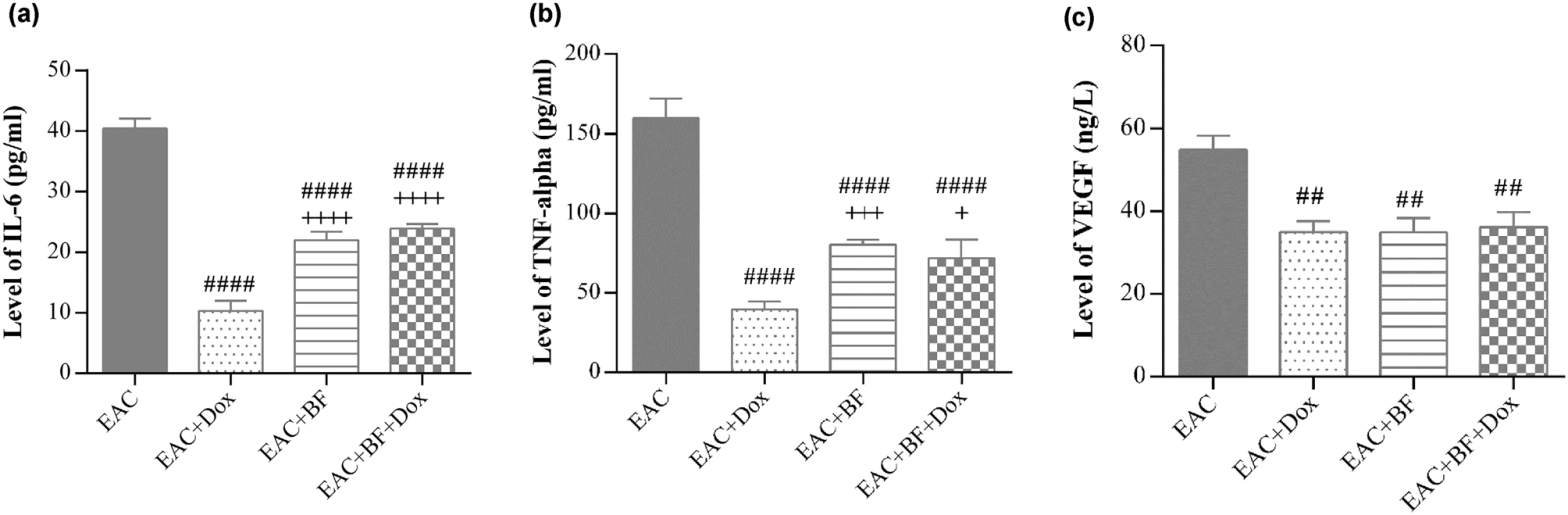
Effects of BF, Dox, and their combination on IL-6, TNF-α, and VEGF levels in EAC-bearing mice. The levels of inflammatory cytokines IL-6 (**a**), TNF-α (**b**), and the angiogenic marker VEGF (**c**) were measured in the ascetic fluid of the untreated EAC group and groups treated with BF (150 mg/kg, orally), Dox (2 mg/kg, i.p.), and their combined mixture after 14 days of treatment. Data are presented as the mean ± SE. Significance values are indicated as follows: “##” and “####” denote *p* < 0.01 and *p* < 0.0001, respectively, compared to the untreated EAC group, and “+”, “++”, and “+++” denote *p* < 0.05, *p* < 0.01, and *p* < 0.001, respectively, compared to the Dox-treated group. BF: galloylquinic acids extracted from* n*-Butanolic fraction; Dox: Doxorubicin; EAC: Ehrlich ascites carcinoma; SOD: Superoxide dismutase; MDA: Malondialdehyde; IL-6: Interleukin-6; TNF-α: Tumor necrosis factor alpha; VEGF: Vascular endothelial growth factor.

A significant decrease was observed in the protein levels of the inflammatory mediators IL-6 and TNF-α (Fig. 5a,b), in the ascitic fluid of all treated groups compared to the untreated EAC group. Among the treated groups, the Dox-treated group exhibited the most pronounced effect. Additionally, Fig. 5c illustrated a marked reduction in the protein level of VEGF in the ascitic fluid of all treated groups compared to the untreated EAC group.

### Effect of BF, Dox, and their combination on liver and kidney function in EAC-bearing mice

**Figure 6:**
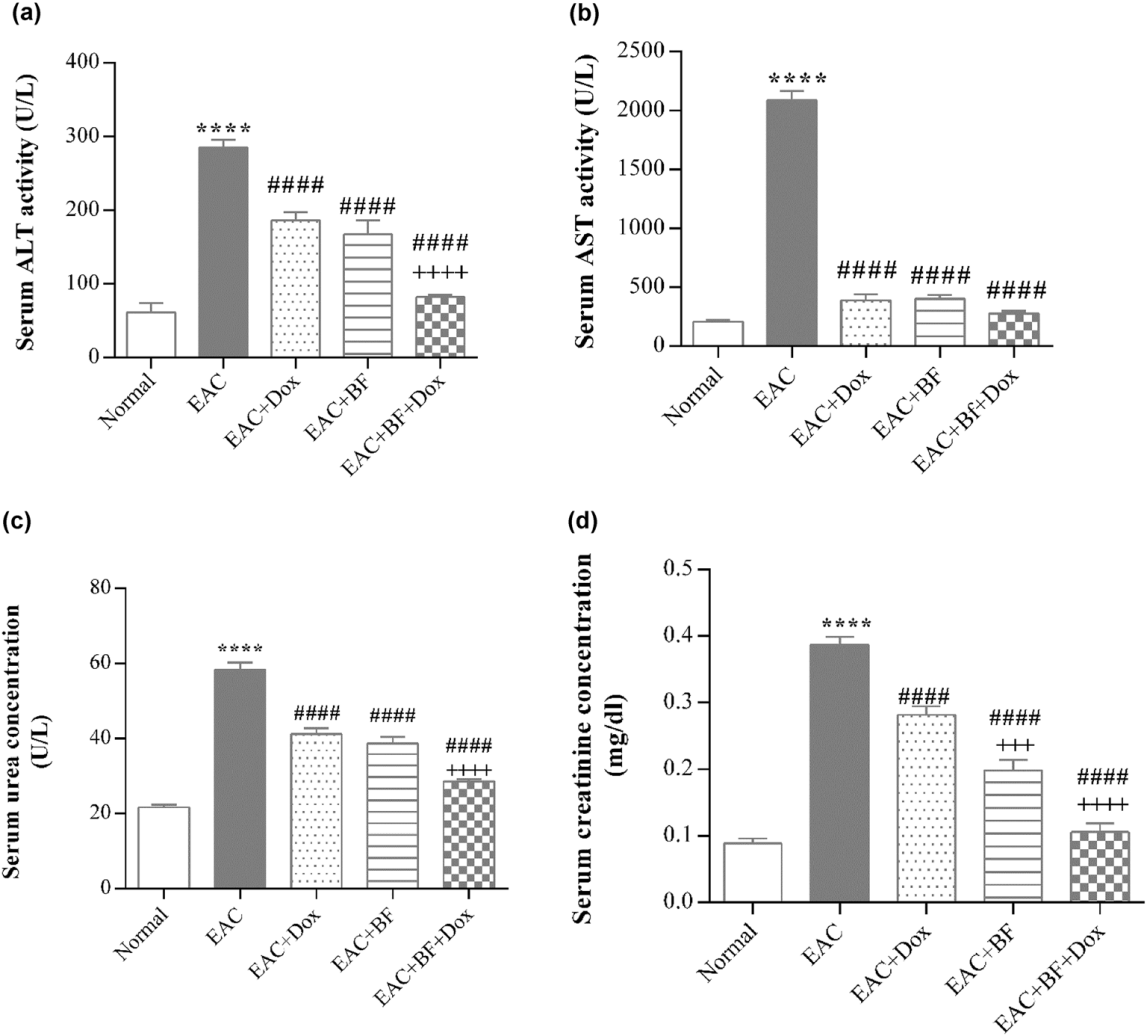
Effects of BF, Dox, and their combination on liver and kidney function in EAC-bearing mice. The serum activities of ALT (**a**) and AST (**b**) enzymes, as well as the levels of serum urea (**c**) and serum creatinine (**d**), were measured in the normal group, untreated EAC group, and groups treated with BF (150 mg/kg, orally), Dox (2 mg/kg, i.p.), and their combined mixture after 14 days of treatment. Data are presented as the mean ± SE. Significance values indicate the following: “****” denotes *p* < 0.0001 compared to the normal group, “####” denotes *p* < 0.0001 compared to the untreated EAC group, and “++++” denotes* p* < 0.0001 compared to the Dox-treated group. BF: galloylquinic acids extracted from* n*-Butanolic fraction; Dox: Doxorubicin; EAC: Ehrlich ascites carcinoma; ALT: Alanine transaminase; AST: Aspartate transaminase.

The examination of liver and kidney function revealed a notable elevation in serum liver enzymes; alanine transaminase (ALT) and aspartate aminotransferase (AST), along with a remarkable increase in levels of kidney function markers, including serum urea and creatinine, in the untreated EAC group. In contrast, all treated groups exhibited significant reductions in ALT activity, serum urea and creatinine levels, with the combination treatment demonstrating the most potent effect compared to the Dox-treated group. These results indicate that BF exhibits a significant synergistic effect when combined with Dox (Fig. 6).

### Effects of BF, Dox, and their combination on histopathological changes in liver, kidney, and lung

**Figure 7:**
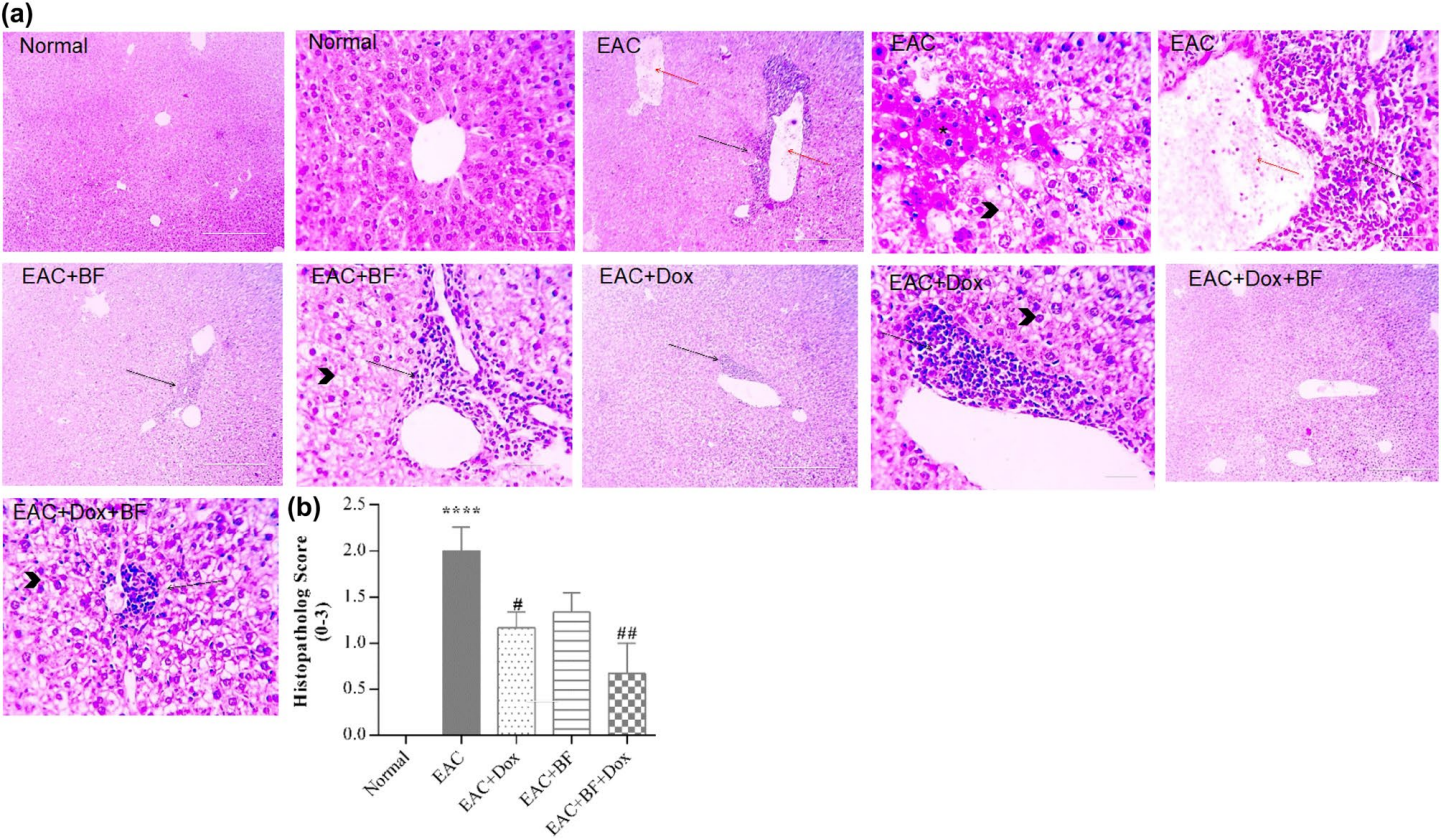
Effects of BF, Dox, and their combination on liver histology. (**a**) Photomicrographs of liver sections from mice in the EAC-mouse model (400 × , H&E stain, scale bar: 50 μm). (**b**) Bar graph showing the effect of BF (150 mg/kg, orally), Dox (2 mg/kg, i.p.), and their combined mixture on the liver injury score compared to the untreated EAC group. Data are presented as the median. Significance values indicate: “****” *p* < 0.0001 versus the normal control group, and “##, ####” *p* < 0.01, 0.0001 versus the untreated EAC group. BF: galloylquinic acids extracted from *n-*Butanolic fraction; Dox: Doxorubicin; EAC: Ehrlich ascites carcinoma; H&E: Hematoxylin and eosin.

The photomicrographs of liver sections stained with H&E in the normal control group revealed the presence of normal hepatic cells arranged in well-organized radially arranged hepatic cords around central veins. Additionally, normal portal areas and sinusoids were observed (Fig. 7a). Meanwhile, liver sections of untreated EAC group showed hydropic degeneration of hepatocytes with occluded sinusoids and multifocal areas of coagulative necrosis, congestion with portal infiltration of EAC cells admixed with mononuclear cells. Liver sections of BF treated group showed diffuse hydropic degeneration, and portal infiltration of EAC cells admixed with mononuclear cells. Similar lesions are detected in Dox treated group.

The BF+Dox-treated group exhibited diffuse hydropic degeneration and portal infiltration of a few mononuclear cells. As shown in Fig. 7b, a significant inhibition of EAC-induced histopathological injury with the combination treatment, followed by the Dox group and then the BF group was observed (Fig. S1).

**Figure 8:**
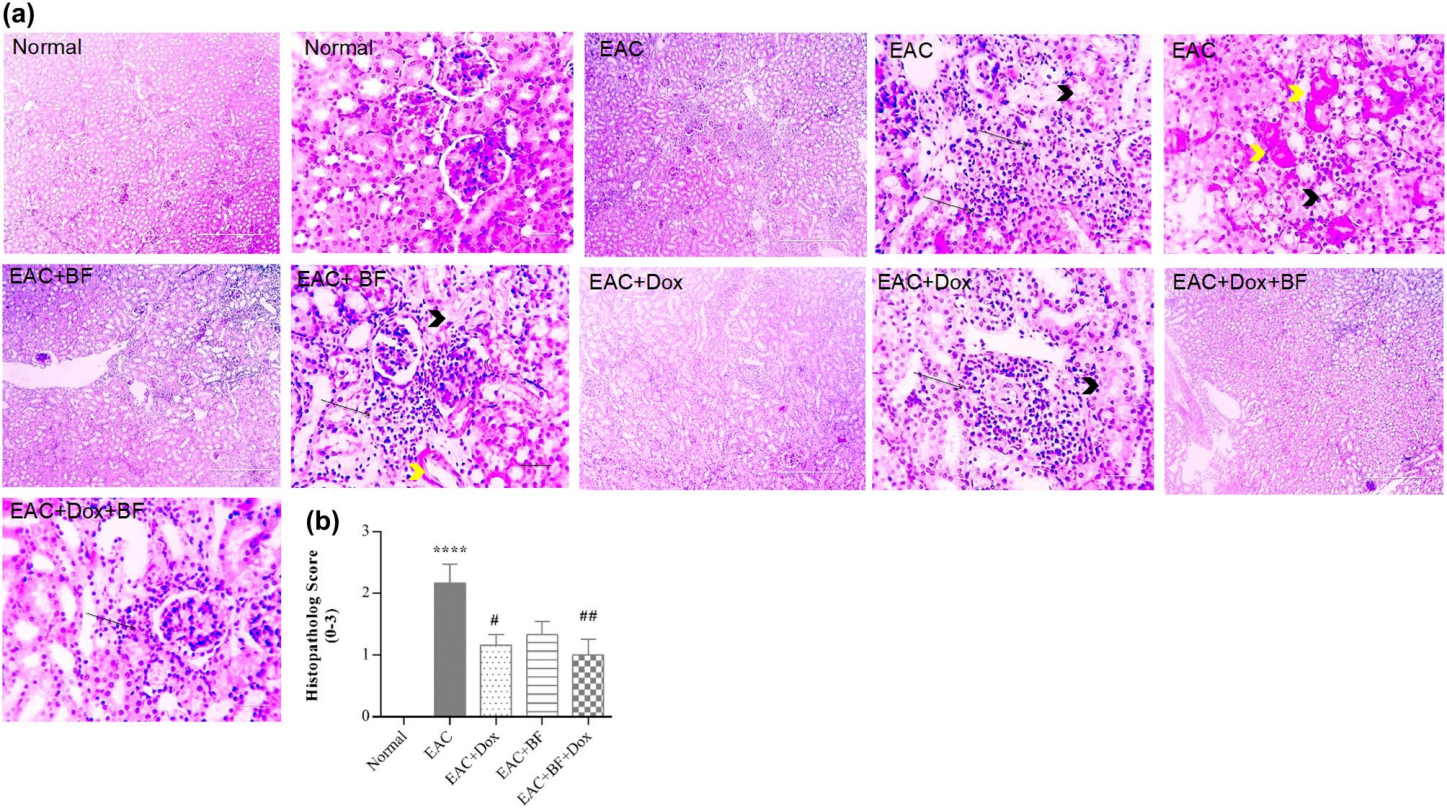
Effects of BF, Dox, and their combination on kidney histology. (**A**) Photomicrographs of kidney sections stained from mice in the EAC model (400 × , H&E stain, scale bar: 50 μm). (**B**) Bar graph showing the effect of BF (150 mg/kg, orally), Dox (2 mg/kg, i.p.), and their combined mixture on the kidney injury score compared to the untreated EAC group. Data are presented as the median. Significance values indicate: “****” *p* < 0.0001 versus the normal control group, and “##, ####”* p* < 0.01, 0.0001 versus the untreated EAC group. BF: galloylquinic acids extracted from *n-*Butanolic fraction; Dox: Doxorubicin; EAC: Ehrlich ascites carcinoma; H&E: Hematoxylin and eosin.

In Fig. 8a, photomicrographs of kidney sections stained with H&E from all mice groups showed normal glomeruli, tubules, and interstitial tissue in the normal group. In contrast, kidney sections of the untreated EAC group exhibited tubular hydropic degeneration and necrosis, accompanied by interstitial infiltration of EAC cells admixed with mononuclear cells. The BF-treated group showed milder tubular hydropic degeneration, necrosis, and interstitial infiltration of fewer EAC cells admixed with mononuclear cells compared to the EAC group. Similarly, the Dox-treated group exhibited mild tubular hydropic degeneration and interstitial infiltration of fewer EAC cells admixed with mononuclear cells. Notably, the (BF+Dox)-treated group displayed minimal infiltration of EAC cells admixed with mononuclear cells in the interstitial tissue.

**Figure 9:**
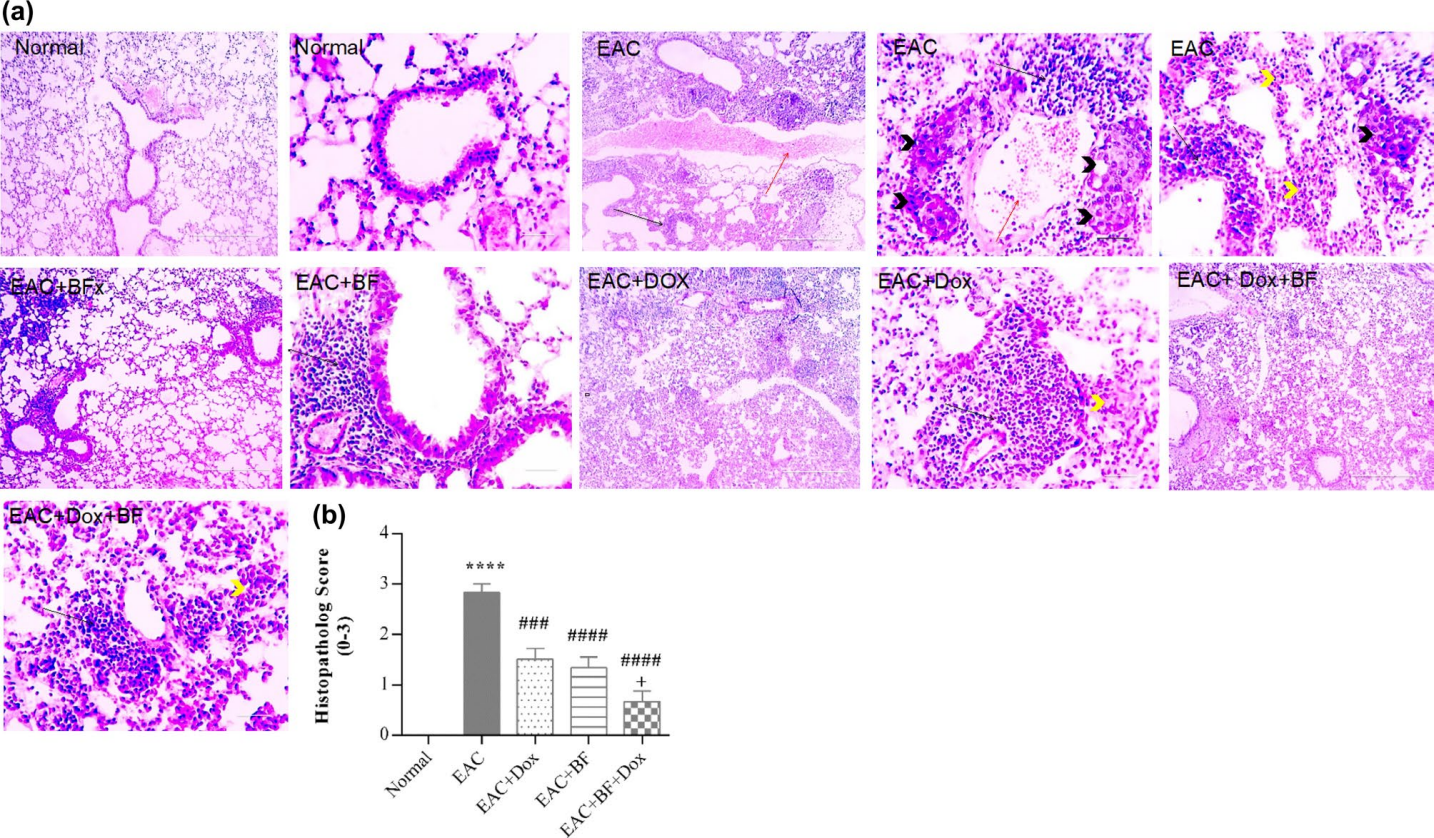
Effects of BF, Dox, and their combination on lung histology. (**A**) Photomicrographs of lung sections from mice in the EAC model (400 × , H&E stain, scale bar: 50 μm). (**B**) Bar graph showing the effect of BF (150 mg/kg, orally), Dox (2 mg/kg, i.p.), and their combined mixture on the lung injury score compared to the untreated EAC group. Data are presented as the median. Significance values indicate: “****” *p* < 0.0001 versus the normal control group, “###, ####” *p* < 0.001, 0.0001 versus the untreated EAC group, and “+” *p* < 0.05 versus the Dox-treated group. BF: galloylquinic acids extracted from *n-*Butanolic fraction; Dox: Doxorubicin; EAC: Ehrlich ascites carcinoma; H&E: Hematoxylin and eosin.

Figure 8b displayed a significant inhibition of EAC-induced histopathological injury with the combination treatment, followed by the Dox group and then the BF group. Photomicrographs of lung sections stained with H&E from all mice groups in Fig. 9a revealed normal bronchioles and alveoli in the normal group. Conversely, the untreated EAC-bearing group exhibited multifocal large areas of tumor cell infiltration around perivascular and peribronchiolar regions, along with the development of solid tumor masses accompanied by vascular congestion and thickening of alveolar walls. The BF-treated group showed perivascular and bronchiolar mononuclear cell infiltration. In the Dox-treated group, perivascular and bronchiolar mononuclear cell infiltration was observed with mild thickening of alveolar walls. The BF+Dox-treated group exhibited minimal perivascular mononuclear cell infiltration and very mild thickening of alveolar walls. Figure 9b demonstrated a significant inhibition of EAC-induced histopathological injury with the combination treatment, followed by the BF group and then the Dox group.

### Cytotoxic effect of BF, Dox and their combination on MCF-7 cells

**Figure 10:**
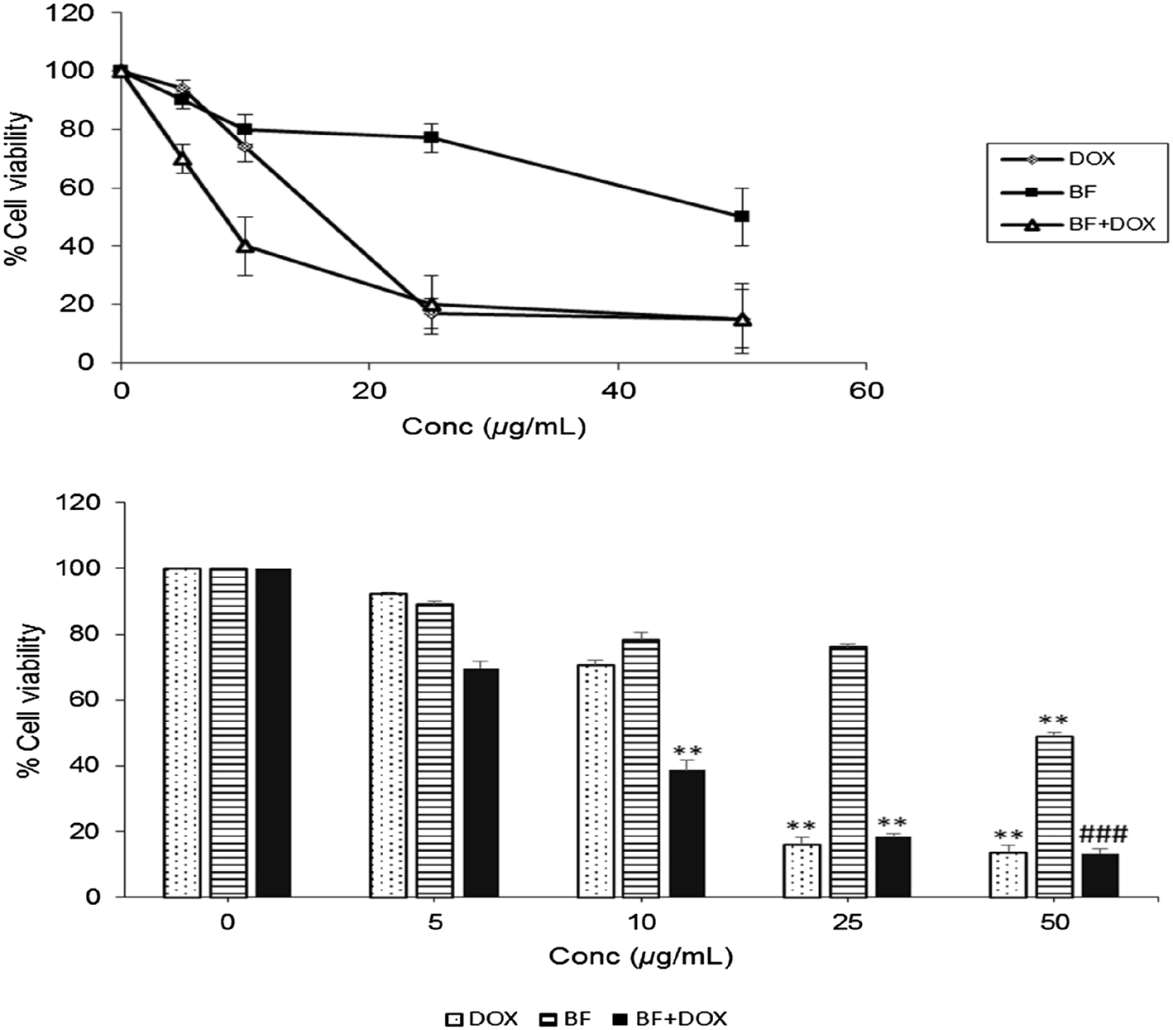
Cytotoxic activity of BF, Dox, and their combination on MCF-7 cell line. Cells were treated with various concentrations of BF, Dox, and their combination (0, 5, 10, 25, 50 μg/mL) for 24 h, and the inhibitory effect was assessed using the MTT assay. Data are presented as mean IC_50_ ± SE. Significance values indicate: “**” *p* < 0.01 versus control, and “###” *p* < 0.001 versus BF: galloylquinic acids extracted from *n-*Butanolic fraction; Dox: Doxorubicin.

Three independent experiments were conducted to determine the IC_50_ values of BF, Dox, and their combination based on the in vitro MTT cytotoxicity data. The obtained IC_50_ values were 50.1 ± 3.4, 17.3 ± 0.9, and 23.4 ± 2.6 µg/mL, respectively. The results demonstrated that BF significantly inhibited MCF-7 cell viability by 48.0 ± 1.0% at a concentration of 50 µg/mL compared to the control untreated cells. Furthermore, the combination of BF with Dox resulted in a significant reduction in cell viability by 38.7 ± 1.5%, 18.3 ± 2.0%, and 13.3 ± 2.0% at concentrations of 10, 25, and 50 µg/mL, respectively. These findings suggest that BF has the potential to enhance the effect of Dox, thereby reducing its required dosage and potential side effects (Fig. 10).

### Modulation of ANXA1 protein level in MCF-7 cells by BF, Dox, and their combination

**Figure 11:**
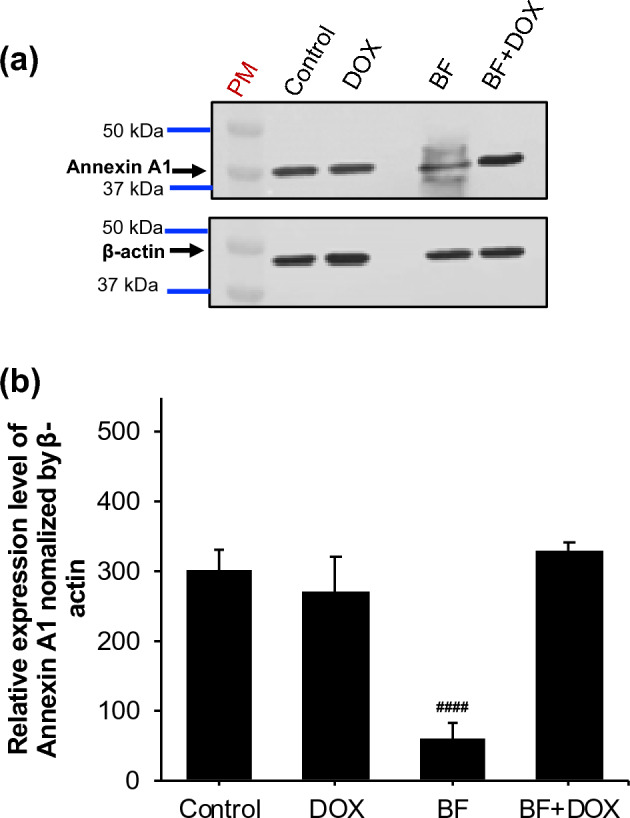
Effect of BF, Dox, and their combination on ANXA1 protein level in MCF-7 cell line. Western blot analysis of MCF-7 cell lysate after 24-h treatment with 50 µg/mL of BF, Dox, and their combined mixture. (**A**) ANXA1 protein expression was assessed by Western blotting using anti-annexin A1 antibody. β-actin was used as the loading control. (**B**) Quantification of ANXA1 bands intensity was performed using Image J software. Each bar represents the mean ± SE from 3 independent experiments. Significance values indicate: “#####” *p* < 0.0001 versus Dox, control, and BF + DOX. BF: galloylquinic acids extracted from *n-*Butanolic fraction; Dox: Doxorubicin; ANXA1: Annexin A1; PM: Protein marker.

Protein levels of ANXA1 were assessed in the MCF-7 cancer cell line using Western blot analysis (Fig. 11a,b). After 24 h of incubation, BF compounds exhibited a significant downregulation of ANXA1 protein level in MCF-7 cells, as demonstrated by the inhibition of protein band intensity compared to control untreated cells and Dox-treated cells (Fig. 11a). However, the combination of BF and Dox did not show any positive effect on ANXA1 levels. This could be attributed to a delayed cellular response or the involvement of distinct mechanisms of action for each drug.

## Discussion

Gallic acid and its derivatives have exhibited selective antitumor effects in various studies. For instance, they have been investigated to reduce biochemical markers associated with skin cancer^29^ and induce cell death in different cancer cell lines, including murine myeloma^30^, leukemia^31,32^, and squamous carcinoma^33^. Another compound, epigallocatechin gallate (EGCG), phytochemical compound, has been reported to facilitate tumor regression in patients with low-grade lymphomas^34^. In the case of galloylquinic derivatives, such as 4,5-di-O-galloylquinic acid, they have demonstrated moderate cytotoxicity specifically against melanoma cells (RPMI-7951)^35^. Extensive research has been conducted on penta-galloyl glucose (PGG), revealing its diverse biological activities associated with cancer therapy and prevention, including antiangiogenic, antiproliferative, anti-inflammatory, and antioxidant properties^36^. However, the use of polyphenols, particularly hydrolysable types like galloylquinic acids, in the treatment of breast cancer is supported by limited research. Hence, in our study, we unraveled the potential synergistic effects of BF compounds extracted from *C. lucens* and Dox on tumor inhibition in EAC-bearing mouse model. To comprehensively assess the combined effects of BF and Dox, we conducted an analysis of tumor size markers, antioxidant capacity, anti-inflammatory activity, antiangiogenic effect, hematological parameters, and histopathological examination of multiple organs function. These analyses were vital in understanding the molecular mechanisms underlying the observed effects and providing further insights into the potential therapeutic implications of BF in combination with Dox for breast cancer treatment. The selection of the EAC model in our study was to explore diverse tumor models and obtain a comprehensive understanding of the therapeutic implications of the tested BF compounds in combination with Dox to further support our previous work using the Ehrlich solid tumor model^37^. By investigating the effect of BF compounds in both the EAC and E solid tumor models, our aim was to provide a robust anticancer activity, to further bolster the efficacy of these BF compounds in breast cancer and investigate their restorative effects on the function of other organs.

The combination of BF and Dox demonstrated an augmented tumor inhibition effect, as evidenced by a reduction in tumor volume and cell count (Table 1). These findings provide insight into the remarkable survival time observed in mice with EAC which were treated with the BF-Dox admixture (Fig. 2). Additionally, by day 15, both the Dox-treated group and the combination-treated group exhibited a significant reduction in the EAC-induced increase in body weight. In contrast, the BF-treated group did not show a significant change in body weight compared to the EAC group (Fig. 3).

Moreover, the combination treatment exhibited a potentiation of the antioxidant defense mechanism (Fig. 4). This was reflected in the inhibition of the lipid peroxidation marker, MDA, indicating a decrease in oxidative damage. Additionally, the activity of the antioxidant enzyme, SOD was elevated. By elucidating the synergistic effects of BF and Dox, our study provides valuable insights into potential combination therapies for breast cancer. The enhanced tumor inhibition, coupled with the reinforcement of the antioxidant defense mechanism, highlights the potential of this admixture as a promising therapeutic approach in the management of breast cancer.

Cancer cells could suppress anti-tumor immunity, through activation of a sustained inflammation that supports tumor growth, metastasis^38,39^ and promote angiogenesis^40^. IL-6 is generated by tumor cells and its signaling is involved in tumor development through increased production of inflammatory cytokines, and growth factors, including VEGF, thus initiated angiogenesis, cell survival and metastasis^41^. TNF-α is a main mediator of inflammatory reactions. Hence, when over-secreted, TNF-α can be an endogenous tumor promoter, and trigger stages of cancer initiation and progression, involving angiogenesis and metastasis^42,43^. Thus, anti-tumor immune response and prevention of neoplastic progression are mediated by cytokine control with anti-inflammatory therapy to reduce over-stimulated pro-inflammatory response, displayed by eicosanoids and cytokines, including TNF-α, and IL-6^44–46^.

TNF-α plays a critical role in the spread of tumors. Within the tumor microenvironment, it stimulates the production of fibroblast growth factor (bFGF), interleukin-8 (IL-8), and vascular VEGF in endothelial cells. The alterations in TNF-α signaling, along with the build-up of MDA (malondialdehyde) in the muscle cell bundles, disrupt cellular integrity and promote tumor growth^20^.

A significant reduction in IL-6 and TNF-α levels was observed in the ascitic fluid of the Dox, BF, and combination treatment groups (Fig. 5a,b). Although the combined treatment did not exhibit an additive anti-inflammatory effect compared to Dox alone, BF demonstrated a remarkable capacity to interfere with cellular and inflammatory signaling pathways. These findings highlight the potential of BF as a promising candidate for future therapeutic advancements in targeting inflammation associated with breast cancer. The ability of BF to modulate inflammatory responses holds promise for the development of innovative treatment strategies aimed at combating the intricate interplay between inflammation and breast cancer progression.

Angiogenesis, as a principle process in cancer development, has appeared to be a possible strategy for cancer medicine^47^. Substantially, growth factors and their related receptors are the regulators of angiogenesis; among these is the VEGF family. VEGF is a cytokine that initiates proliferation, migration, and permeability^48^. Its role in breast cancer angiogenesis, has been an fascinating field of investigation^49^. Previous studies proposed that under pathological conditions, TNF-α promoted cellular angiogenic activity through its capability to induce transcriptional activation of VEGF and its receptors, leading to cancer initiation. The therapeutic benefit of angiogenesis inhibition by decreasing cytokine levels was reported in in vivo and in vitro models^50–54^. Consequently, the role of VEGF as a therapeutic target in breast cancer treatment has motivated our investigation into the potential of BF compounds derived from *C. lucens* as novel anticancer agents. The administration of Dox, BF, and their combination exhibited a pronounced antiangiogenic effect, as evidenced by the significant reduction in ascitic VEGF levels (Fig. 5c). This antiangiogenic activity aligns with the overall anti-inflammatory effects observed with all treatments. Notably, our findings suggest that BF, both alone and in combination with Dox, hold promise as a valuable adjunct to existing anti-angiogenic therapies. The ability of BF to modulate angiogenesis provides a compelling rationale for further exploration of its potential in enhancing and complementing current treatment strategies aimed at inhibiting tumor-associated angiogenesis in breast cancer.

The current treatment of breast cancer includes: surgery, chemotherapy, radiation therapy, hormonal therapy, targeted therapy and immunotherapy, however these interventions cause severe side effects to most women, which are problems when treatment affects healthy tissues or organs. Additionally, multiple drug resistance is a major cause of unsuccessful chemotherapy^55^. Diverse natural compounds such as the flavone Chrysin, bee venom, and melittin could reduce drug resistance, and given as adjunct to chemotherapeutic drugs or radiotherapy to improve their efficacy^19,20,56^. Galloylquinic acid compounds from *Copaifera* species, are natural phytochemicals, confirmed to have cytoprotective effects against gastric^11^, colorectal^12^, and solid^37^ tumors, supporting their potential as therapeutic target in breast cancer, and Doxorubicin-associated resistance.

Several studies have established a link between breast cancer and liver inflammation and fibrosis. Breast cancer can have systemic effects on various organs in the body, and the liver is particularly vulnerable to these metastatic effects. When breast cancer spreads to the liver, it can lead to the activation of inflammatory processes, cytokines release and the development of fibrosis. This can impair liver function and compromise its ability to perform detoxification and metabolism^57^.

The inflammatory mediator TNF-α has the capacity to induce the expression of E-selectin in endothelial cells, including those present in the liver sinusoids. Studies have revealed that breast cancer cells possess the capability to initiate an inflammatory cascade, leading to increased adhesion to liver sinusoidal endothelial cells, a phenomenon observed in lung cancer as well. Although the process of tumor cell attachment to the endothelium during metastasis is complex and multifactorial, the production of TNF-α-induced endothelial E-selectin by tumor cells appears to represent a pivotal stage in the progression of breast cancer liver metastasis^58^. In a study conducted by Asgeirsson et al.^59^ it was observed that the induction of IL-6 led to a decrease in cell adhesion across three breast cancer cell lines, concomitant with a reduction in E-cadherin expression. Additionally, patients with liver metastases from breast cancer exhibited notably elevated levels of IL-6. These findings suggest that breast cancer cells establish a pro-inflammatory microenvironment by releasing various cytokines, which in turn promote the adhesion and invasion of tumor cells into the liver. The rapid division of EAC cells in the peritoneal cavity leads to increased tumor cell count and volume, creating a hypoxic environment nearby. This triggers the release of angiogenic growth factors and cytokines such as VEGF and TNF-α, activating endothelial cells. As new capillaries form, these angiogenic factors and cytokines enter the portal circulatory system, eventually reaching the liver through the portal vein. They bind to specific receptors, initiating various signal transductions. Consequently, hepatic stellate cells, Kupffer cells, and mast cells are activated, contributing to hepatic inflammation, fibrosis, and liver injury^57^.

BF compounds exhibited complementary beneficial effects on the function of multiple organs, including the liver and kidney, by modulating serum ALT and AST enzymes activities, as well as the levels of serum creatinine and urea (Fig. 6). In addition to their potential impact on maintaining the normal histological characteristics of the liver, kidney, and lung (Figs. 7, 8 and 9). BF compounds also played a significant role in modulating blood counts normal values, including Hb levels, RBC count, and WBC count (Table 2).

Our study also encompassed in vitro experiments to investigate the potential synergistic effects of BF compounds with Dox on cytotoxicity in MCF-7 cells. The experimental approaches employed MTT analysis to assess cell viability, as well as Western blot analysis for ANXA1 level. BF compounds significantly inhibited the viability of breast cancer cells in a dose-dependent manner alone and in combination with Dox (Fig. 10). In the Western blot analysis, the band intensities of ANXA1 relative expression in MCF-7 cells were similar between the control untreated cells and cells treated with Dox alone, suggesting that Dox alone did not significantly affect ANXA1expression levels (Fig. 11). However, when the cells were treated with our tested BF compounds, there was a significant decrease in ANXA1 band intensity. Regarding the combination of BF and Dox, the band intensities remained similar to the control untreated cells, suggesting that the presence of Dox did not further influence the decrease in ANXA1expression caused by BF. This could indicate that the effects of BF on ANXA1expression were dominant or not influenced by the presence of Dox in the combination treatment. Several factors could potentially explain these observations, such as differential mechanisms of action in which BF and Dox might have different mechanisms of action in modulating annexin A1 expression. BF may directly or indirectly downregulate ANXA1 expression, while Dox might not have a significant impact on ANXA1 levels in MCF-7 cells. It is also possible that the combination of the tested BF and Dox could result in synergistic or antagonistic effects on ANXA1 expression. In this case, the combination may have counteracted the decrease in ANXA1 caused by our compounds BF, resulting in similar band intensities compared to the control untreated cells.

The precise mechanisms and triggers of ANXA1 expression in breast cancer are still being investigated. Further research is warranted to fully elucidate the complex regulatory networks involved in ANXA1 activation. Understanding these regulatory mechanisms holds promise for the development of targeted therapies aimed at modulating ANXA1 expression and improving breast cancer treatment outcomes^60,61^. One study showed that triple-negative breast cancer characterized by high Annexin A1 expression and lacking hormone receptor expression, is linked to the presence of mast cells, inflammatory response, and angiogenesis^62^.

The expression of ANXA1, is influenced by various factors in breast cancer. There are some known factors associated with the activation of ANXA1 expression and their implications in breast cancer. Inflammatory cytokines, such as IL-6 and TNF-α, have been found to upregulate ANXA1 expression in breast cancer cells. These cytokines, commonly present in the tumor microenvironment, activate specific signaling pathways that promote ANXA1 expression^62–64^. Hormonal signaling also plays a role in ANXA1 regulation. Estrogen, a pivotal hormone in breast cancer development, is implicated in the modulation of ANXA1 expression. Estrogen receptor signaling can directly or indirectly modulate ANXA1 levels in breast cancer cells^65,66^.

**Figure 12:**
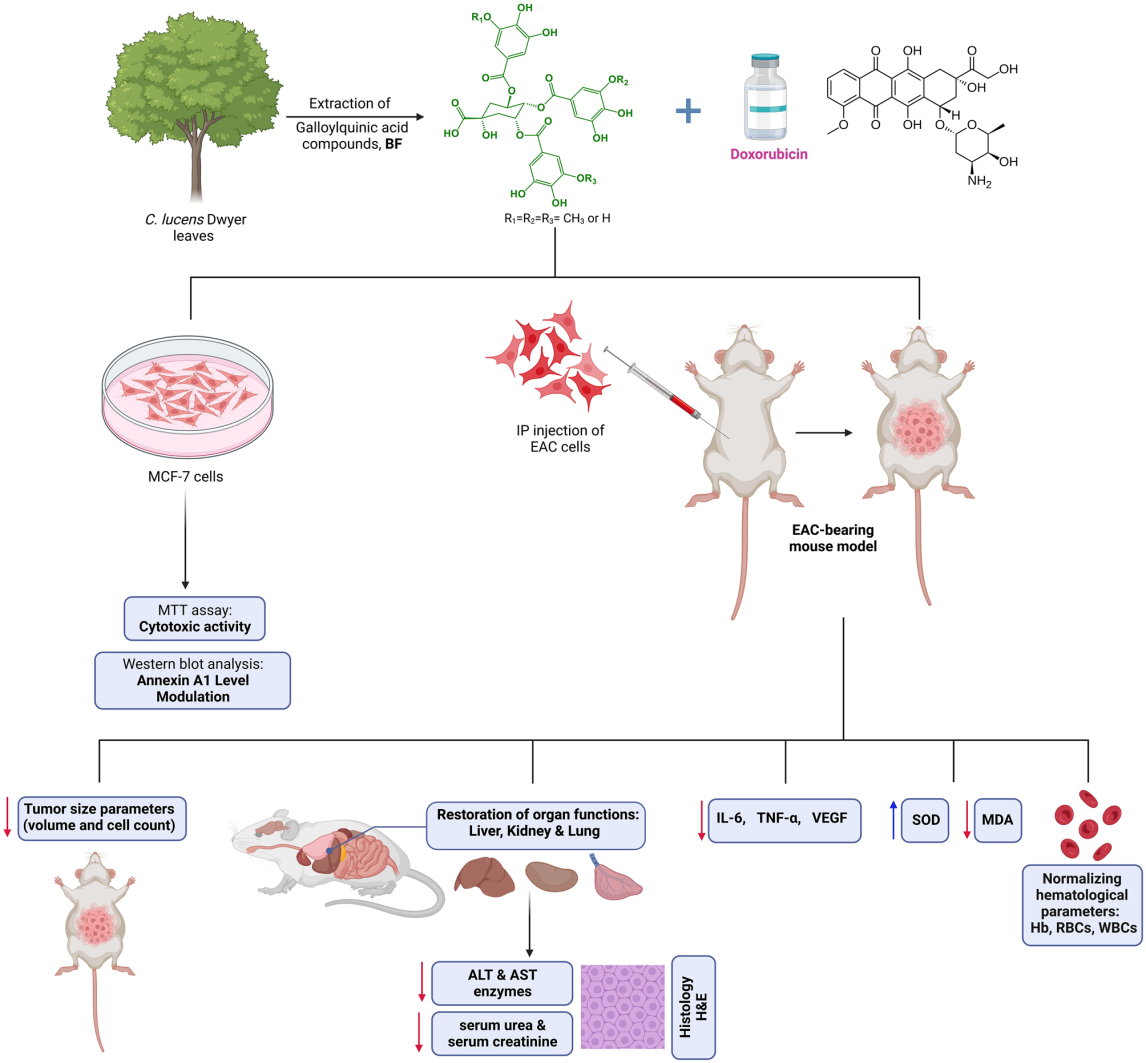
Comprehensive overview of the potential effects and mode of action of galloylquinic acid compounds and their combination with doxorubicin in breast cancer models.

It has been also reported that ANXA1 can behave as both an oncogene and tumor suppressor^67,68^ with differentiated expression levels dependent on cell, tissue, and cancer types. ANXA1 is an immune-modulating protein with diverse functions in immune system, cancer, central and peripheral inflammation. ANXA1 has also multiple effects beyond the immune system with implications in maintaining the homeostatic environment within the entire body, due to its ability to affect cell–cell signaling, hormonal secretion, fetal development, the aging process and development of a disease. Therefore, ANXA1 modulation, as a potential pharmacological protein has been linked to the therapeutic potential of a wide range of cancers, such as breast cancer^66,69–71^. Contrarily, other studies indicated that high ANXA1 expression is positively correlated with disease severity, increasing tumor stage^72^, and enhancing migration, through constitutive initiation of NF-κB^73^, and its downstream extracellular signal-regulated kinases (ERK) activation^74^, in addition to transforming growth factor beta (TGF-β)-dependent signaling promotion^75^. However, further studies are required to understand the exact mechanisms of ANXA1-mediated cellular activation. To our knowledge, no previous investigations have explored the role of BF as potential modulators of annexin ANXA1 in cancer models, specifically concerning the progression of breast cancer. Therefore, our study presents novel insights into the therapeutic potential of BF by highlighting their unexplored interaction with ANXA1, thus contributing to a deeper understanding of the molecular mechanisms underlying breast cancer progression. The overall findings of this study may pave the way for future research aimed at harnessing the therapeutic benefits of BF in the context of ANXA1-mediated pathways in cancer (Fig. 12).

## Methods

### Material

Doxorubicin was purchased from Pfizer (Egypt), Trypan Blue was obtained from Sigma-Aldrich (St. Louis, MO, USA). All other chemicals used in the study were of high analytical purity. Galloylquinic acids compounds were extracted from the *n*-butanolic fraction of *C. lucens* following the methodology described in our previously reported studies^10,37^.

### Cell lines

Ehrlich ascites carcinoma cells (EAC) were obtained from the National Institute of Cancer, Cairo, Egypt. Fresh EAC cells were maintained in mice by serial intra-peritoneal (i.p.) injections of 1 × 10^6^ cells. After seven to ten days, ascitic fluid containing EAC cells was collected from the peritoneal cavity using a needle, and cell viability was assessed by trypan blue staining (A cell viability threshold of 95% or higher was considered as the accepted standard). Cell viability percentage was calculated using the following equation:

$$Viability\, \left(\%\right)\,of \,EAC\, cells = \frac{No. \,of \,viable\, cells}{Total \,No. \,of\, viable \,and \,dead\, cells} \times 100$$

Subsequently, the viable tumor cells were resuspended in phosphate-buffered saline (PBS) and centrifuged at 1500 rpm for 10 min^1,76^. For the in vitro assays, MCF-7 cells (ATCC HTB-22™) were utilized. The aliquot of these cells is courtesy by IRB Barcelona, Spain.

### Experimental animals and ethics

Animals were handled and all experimental procedures were conducted in accordance with the Animal Research: Reporting of In Vivo Experiments (ARRIVE) guidelines^77^, and in compliance with the instructions of the Institutional Animal Care and Use Committee (IACUC), which was approved by the Faculty of Pharmacy, Delta University for Science and Technology (FPDU# 151121/13). Sixty female Swiss albino mice (20–25 g) aged approximately 8 to 10 weeks were housed at the animal facility of the Faculty of Pharmacy, Delta University for Science and Technology. The mice were kept in cages under controlled conditions, including a 12-h light/dark cycle, suitable temperature (20 ± 5 °C), and 65% relative humidity. They were provided with free access to standard food and water throughout the seven-day acclimatization period prior to the start of the study, which commenced with the i.p. inoculation of EAC cells into the animals.

### Ehrlich ascites carcinoma (EAC) model induction and animal treatment

For tumor induction, mice were inoculated with 1 × 10^6^ EAC cells each (0.2 mL/mouse, i.p.), then randomly divided into four groups (n = 12), 2 h after inoculation. Experimental groups were treated daily with Dox (2 mg/kg, i.p.)^76,78^ and BF (150 mg/kg, orally)^11,79^ for 14 days^76^, according to the following scheme: Group I-Normal group (without EAC cells inoculation); PBS was injected i.p. daily for 14 days. Group II-EAC group; 1 × 10^6^ EAC cells were previously injected (0.2 ml/mouse, i.p.). Group III-EAC + Dox treated group; 1 × 10^6^ EAC cells were previously injected, then animals were treated daily with Dox at a dose of (2 mg/kg, i.p.) for 14 days. Group IV-EAC + BF treated group; 1 × 10^6^ EAC cells were previously injected, then animals were treated daily with BF at a dose of (150 mg/kg) orally, for 14 days. Group V-EAC + BF + Dox combination group; 1 × 10^6^ EAC cells were injected, then animals were treated daily with a combination of BF and Dox at respective doses of (150 mg/kg, orally) and (2 mg/kg, i.p.) for 14 days. Body weight changes were recorded every 5 days from day 0 to day 15. Twenty-four hours after the last dose, blood was collected via retro-orbital puncture from all groups for further hematological and biochemical analyses. Euthanasia was performed using inhalation anesthesia by placing animals in a closed chamber with 5% isoflurane. The ascetic fluid containing tumor cells was collected from peritoneal cavity of six mice from groups II–V. Antitumor effects were assayed in the ascitic fluid by immediately determining tumor size markers, including ascitic tumor volume, viable and non-viable tumor cell counts, in the treated groups compared to the control EAC group. The remaining mice in Groups II–V were retained alive to determine the mean survival time (MST). Ascetic fluid samples were suspended in PBS, and then centrifuged at 1800 rpm for 10 min at 4 °C^4,76^. The pellets were frozen in liquid nitrogen, and then stored at − 80 °C until further use in immunoassays.

### Hematological and biochemical assessment

Blood was collected on day 15 from all groups in tubes containing EDTA (for hematological analysis) and without anticoagulant (for serum biochemical assays). Hematological analysis for Hb, RBCs and WBCs counts, were performed utilizing standard automated procedures^80^.

Serum was obtained by centrifugation of blood at 4000 rpm for 10 min at 4 °C. Biochemical analysis was performed using ALT and AST colorimetric assay kits (SPINREACT, Sant Esteve de Bas, Spain) for the assay of liver function. Creatinine and blood urea nitrogen (BUN) colorimetric assay kits (Biomed diagnostic, Egypt), were used as markers for kidney function. Moreover, the assessment of oxidative stress was performed through detection of lipid peroxidation and antioxidant status using MDA, and superoxide dismutase SOD colorimetric assay kits, respectively, obtained from (Biodiagnostic company, Egypt). All measurements were executed according to the manufacturer instructions.

### Determination of inflammatory cytokine levels (TNF-α and IL-6)

Inflammatory cytokine levels in the ascetic fluid were measured using sandwich enzyme-linked immunosorbent assay (ELISA) kits specific to mouse TNF-α and IL-6. The ELISA kits, purchased from Innova Biotech Co., Ltd., China (Cat # In-Mo1920 for TNF-α and In-Mo1283 for IL-6), were used for the assessment of cytokine levels. The protocol followed the manufacturer instructions, which involved incubating the samples and reagents in the ELISA plates for 30 min at 37 °C, followed by washing. Subsequently, horseradish peroxidase conjugated antibodies for mouse TNF-α and IL-6 were added, and further incubated before washing. The final steps included coloration, termination, and measuring the absorbance at 450 nm. The concentrations were expressed in pg/mL.

### Determination of vascular endothelial growth factor (VEGF) level

The levels of VEGF, a key regulator of angiogenesis, were determined in the ascetic fluid using a mouse VEGF ELISA kit obtained from Innova Biotech Co. Ltd., China (Cat# In-Mo1806). The assay procedure was carried out following the manufacturer instructions, as mentioned previously, and the VEGF concentrations were expressed in ng/L.

### Histopathological findings

For histological analysis, the liver, kidney, and lung samples were immediately fixed in 10% neutral buffered formalin (pH 7.2) and subsequently dehydrated through a series of graded alcohol solutions. The samples were then embedded in paraffin. Thin sections of 5 µm thickness were cut from the paraffin-embedded tissues. These sections were stained with Hematoxylin and Eosin (H&E) stain. The prepared slides were examined under a light microscope, and images were captured using a digital camera. The assessment of tissue abnormalities was conducted in a blinded manner, and the observed structural changes were semi-quantitatively graded on a scale from 0 (indicating a normal structure) to 3 (representing severe pathological changes). This grading system facilitated a comparative analysis of the extent of structural alterations^81^.

### Determination of cell viability by MTT assay in MCF-7 cells

The in vitro anticancer potential of BF, Dox or their combined form was evaluated by MTT colorimetric assay (Cell Titer 96^®^ Non-Radioactive Cell Proliferation Assay, Promega Biotech Ibérica S.L.) according to a previous method^82^. The cells of MCF-7 were cultured in 75-mL flask, once confluent, the cells (2 × 10^4^ cells per well) were seeded onto 96-well microplate and treated with various concentrations of BF, Dox and their mixture (0, 5, 10, 25, 50 μg/mL in 0.1% dimethyl sulfoxide (DMSO) diluted in phosphate buffered saline (PBS)) for 24 h. The working solution of MTT was added to each well and incubated at 37 °C for 3 h, followed by stop solution addition (100 μL). The optical density (OD) was measured at 570 nm using a microplate reader (Gemini XPS and EM Microplate Reader, USA). Cell viability was calculated as a percentage of viable cells in compound- treated group versus untreated control using the following equation:

$$Cell \,viability \,\left(\%\right)=\left[\frac{OD\, \left(test\right)}{OD \,\left(control\right)} \right]\times 100$$

### Western blot analysis of Annexin A1 level in MCF-7 cells

MCF-7 cells (5 × 10^5^ cells/well) were seeded in a 6-well plate and cultured overnight in complete DMEM medium supplemented with 10% fetal bovine serum (FBS). Following this, the cells were treated with 50 μg/mL of Dox, BF, or their combination for 24 h. After treatment, the cells were washed with PBS and then treated with ice-cold whole cell lysis buffer containing Protease inhibitor (ROCHE cOmplete™ Protease Inhibitor Cocktail, tablets). The cells were gently scraped, collected, and incubated with the lysis buffer on an ice bath for 30 min with intermittent agitation every 10 min. The lysed cells were centrifuged at 10,000 rpm for 12 min, and the protein supernatant was collected and kept on an ice bath. The protein concentrations were determined using the Bradford method with the Pierce BCA Protein Assay Kit from Thermo Scientific. For gel electrophoresis, 30 μg of proteins were mixed with Laemmli sample buffer (1:1) from Bio-Rad and boiled at 99 °C for 5 min. The proteins were then separated on a 7.5% ready gel (Bio-Rad) by running at 120 V for 120 min. After gel electrophoresis, the proteins were transferred onto a nitrocellulose membrane using 1 × transfer buffer. The membrane was probed with our previously characterized^82^ rabbit polyclonal anti-ANXA1 (1:1000) and mouse monoclonal anti-β-actin (1:1000) antibodies (Cell Signaling, USA) in blocking buffer, separately, and incubated at 4 °C overnight. After washing the membrane three times for 5 min each with TBS/0.1% Tween, it was incubated with the appropriate secondary antibody conjugated with horseradish peroxidase (1:5000) (Rabbit IgG horseradish for ANXA1 antibody or mouse IgG horseradish peroxidase for β-actin antibody) (Cell Signaling, USA). The incubation was carried out in 1 × Tris-buffered saline/0.1% Tween 20 and 5% milk for 1 h at room temperature. The immunoreactive bands were detected by developing membranes with SuperSignal™ West Femto Maximum Sensitivity Substrate (Thermo Scientific, USA). The bands were then visualized using autoradiography. The intensity of each band was quantified using ImageJ software.

### Statistical analysis

The data values were presented as mean ± standard error, and the graphs were generated using Graph Pad Prism 6 software (Graph Pad, San Diego, CA). Statistical analysis was performed to assess significant differences between various groups. One- and two-way ANOVA (analysis of variance) followed by Tukey–Kramer's multiple comparison test were utilized for comparisons involving multiple groups. Additionally, unpaired Student's *t*-test was employed for comparisons between two groups. Statistical significance was defined as *p* < 0.05.

## Conclusion

Our study demonstrates that BF compounds exhibit potent in vivo antitumor effects in an EAC model. These compounds not only contribute to the normalization of blood count parameters but also show positive effects on hepatic, renal, and pulmonary histology in EAC-bearing mice. Moreover, they effectively restore liver and kidney functions while displaying antioxidant defense mechanisms. Additionally, the combination of BF compounds with Dox reveals promising anti-inflammatory and anti-angiogenic properties. Furthermore, these compounds exhibit cytotoxic effects and significantly decrease the level of ANXA1 in MCF-7 cells. Overall, our findings suggest that BF compounds hold great promise for future therapeutic advancements in breast cancer modulation. They have the potential to enhance Dox sensitization, improve efficacy, decrease non-specific cytotoxicity, and potentially overcome resistance commonly associated with cancer treatment.

## Supplementary Information

Supplementary Information.

## Data Availability

The data supporting the findings of this study are available from the corresponding authors upon reasonable request.
