# Supplementary material for: Suppression of VEGF and inflammatory cytokines, modulation of Annexin A1 and organ functions by galloylquinic acids in breast cancer model
Source: Sci Rep. 2023 Jul 28;13:12268. doi: 10.1038/s41598-023-37654-4 (PMC10382581; doi:10.1038/s41598-023-37654-4)
Supplement: Supplementary file 1 — Supplementary Information. [file 41598_2023_37654_MOESM1_ESM.pdf]

Suppression of VEGF and inflammatory cytokines, modulation of Annexin A1 and organ functions by Galloylquinic acids in breast cancer model

Mohamed Abd El-Salam[1,*], Ghada El-Tanbouly[2], Jairo Bastos[3] and Heba Metwaly[4*]

[1]*Department of Pharmacognosy, Faculty of Pharmacy, Delta University for Science and Technology, Gamasa, 11152 Egypt*

[2]*Department of Pharmacology, Faculty of Pharmacy, Delta University for Science and Technology, Gamasa, 11152 Egypt*

[3]*Department of Pharmaceutical Sciences, School of Pharmaceutical Sciences of Ribeirão Preto, University of São Paulo, Ribeirão Preto, São Paulo, 14040-900, Brazil*

[4]*Department of Pharmaceutical Biochemistry, Faculty of Pharmacy, Alexandria University, Alexandria, 21500, Egypt*

Supplementary Information (SI):

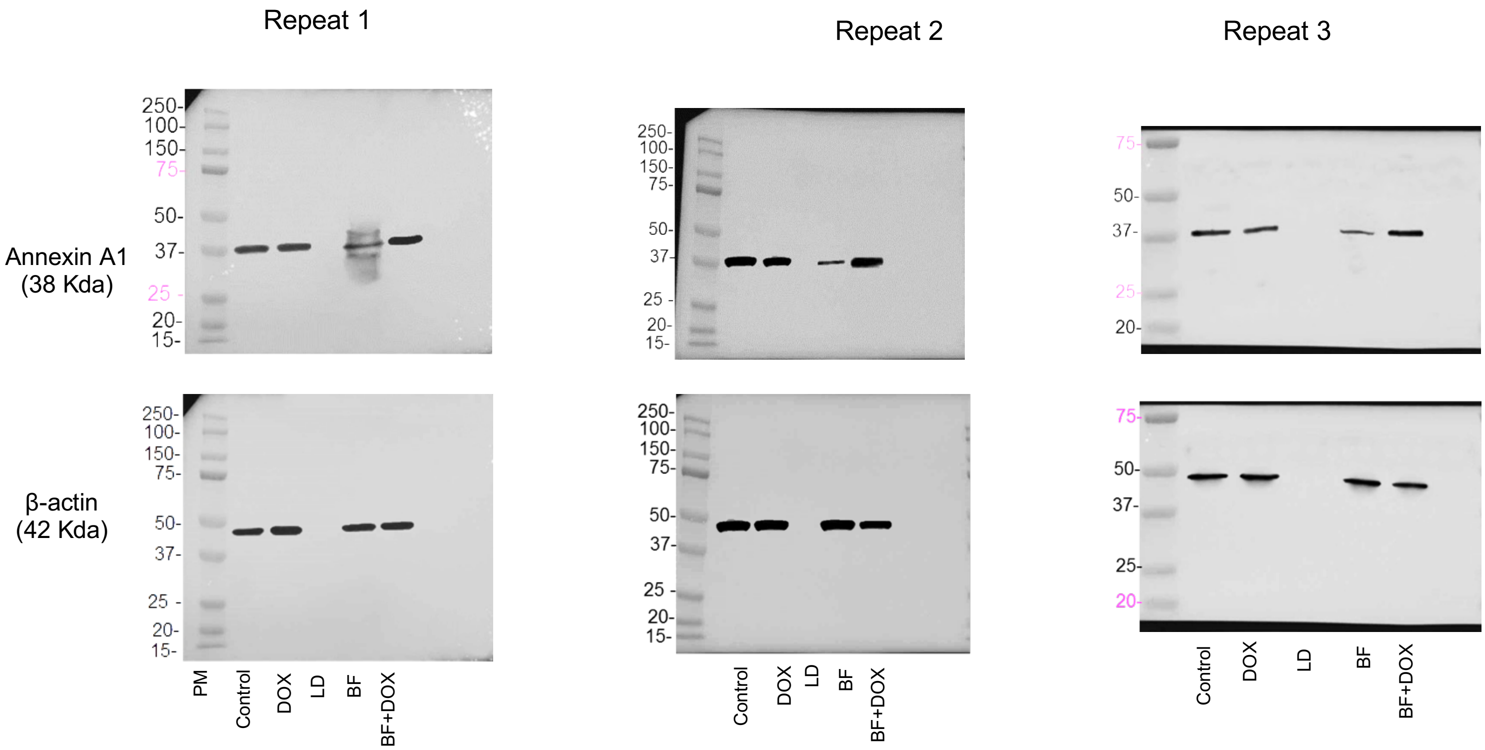

Fig. S1. Effect of BF, Dox, and combination treatment on ANXA1 protein level in MCF-7 cell line. The figure shows whole western blots of MCF-7 cell lysates after treatment with 50 µg/mL of galloylquinic acid compounds (BF), doxorubicin (Dox), and their combined mixture for 24 hours. The proteins extracted from the whole cell lysate were subjected to Western blotting analysis using an anti-annexin A1 antibody (Cell Signaling #3299), with β-actin serving as the loading control. The images were captured using the automated GE Healthcare - Amersham Imager 600 Series under consistent contrast conditions for all images. The antibodies used in the experiment have been previously characterized and reported in our study[1]. PM: protein marker; LD: loading dye.

1. Abd El-Salam, M. et al. The Synthesized Plant Metabolite 3,4,5-Tri-O-Galloylquinic Acid Methyl Ester Inhibits Calcium Oxalate Crystal Growth in a Drosophila Model, Downregulates Renal Cell Surface Annexin A1 Expression, and Decreases Crystal Adhesion to Cells. 61, 1609-1621, doi:10.1021/acs.jmedchem.7b01566 (2018).
